# “It’s like fighting a war with rocks”: Nursing home healthcare workers’ experiences during the COVID-19 pandemic

**DOI:** 10.1017/ice.2020.393

**Published:** 2020-08-05

**Authors:** Sandhya Seshadri, Cathleen Concannon, Jane A. Woods, Kathryn M. McCullough, Ghinwa K. Dumyati

**Affiliations:** 1 School of Nursing, University of Rochester, Rochester, New York; 2 Department of Neurology, University of Rochester, Rochester, New York; 3 Center for Community Health and Prevention, University of Rochester Medical Center, Rochester, New York; 4 Infectious Diseases Division, University of Rochester Medical Center, Rochester, New York

*To the Editor—*The coronavirus disease 2019 (COVID-19) pandemic has resulted in unprecedented stress and has revealed significant vulnerabilities in nursing homes. They lack resources such as adequate staffing,^1,2^ financial reserves to address unexpected expenses, and physical spaces to contain the spread of the highly contagious novel coronavirus. These shortages have impacted the care of vulnerable older adults and the physical and emotional well-being of healthcare workers (HCWs). As part of a larger study on HCW COVID-19–related risks and exposures, we interviewed 161 nursing home staff from 28 nursing homes who tested positive for severe acute respiratory syndrome coronavirus 2 (SARS-CoV-2). The study underwent research ethics review and approval. Herein, we report on nursing home HCW experiences, and we recommend measures to better support them.

The pandemic worsened the critical problem of chronic understaffing in nursing homes^3^ due to absenteeism and infected staff who were quarantined. Historically, nursing homes hired temporary nurses from agencies to manage staffing needs, but during the pandemic, some “agency nurses just bolted.” Available HCWs were taxed to the limit (Table 1, Q1 and Q2). As one physical therapist noted, “I do everything… I clean residents, feed them, take blood pressure—you name it.” With the lack of readily available space to cohort infected residents, containing the infection has been difficult. Residents sometimes live in a facility for years, and moving them to units dedicated to the care of those infected is challenging because it entails moving all their belongings, too. Nursing homes attempted to cohort staff by color coding units as green, yellow, and red, with “green units” being free of COVID-19 residents. Due to staffing shortages, nurses “floated” from one unit to another, putting other residents at risk (Table 1, Q3 and Q4).

**Table 1.** Quotations That Exemplify Nursing Home Staff’s Experiences with Caring for Residents with COVID-19

Quotations
Q1 They wouldn’t let facilities come to clean the rooms because of isolation. So, I had to do everything. A resident has diarrhea and we are cleaning them, changing their clothes, cleaning the room. You name it, I have done it. (RN)
Q2 I am tired. I am exhausted. I have been taking care of COVID patients since March. I called in for a “personal day” on Wednesday because I was tired. But they don’t care. (CNA)
Q3 We had agency workers floating to all floors and weren’t wearing face shields. (LPN)
Q4 I was told that I was working on a “green unit,” but they had me floating to “yellow” units all the time. (LPN)
Q5 We wore the same gown for an entire shift. When there weren’t enough gowns, I wore a lab coat. I am 5’3”, 140 pounds, and the lab coats were too big. The top came down to my sternum, so I had to wear it backwards. As you can imagine, it was difficult to wear while taking care of patients. (Activities aide)
Q6 Once there were confirmed cases, there was no education on where we should be donning and doffing PPE. There was a lot of misinformation and confusion floating around. It was incredibly disorganized. We did not know when we should be cleaning equipment and where we should be cleaning. (CNA)
Q7 It was so confusing. Every day it [PPE requirements/rules] was different. You never knew for sure. It was hard to decipher. (CNA)
Q8 I don’t understand. I did everything right. How did I get the virus? (CNA)
Q9 I am extremely concerned about going back to work and catching this again. (LPN)
Q10 I had to protect my family. (Physical therapist)
Q11 We couldn’t get surgical masks. They give you only 1 mask and that is ridiculous…I put myself at risk, I work 12-hour shifts and I did everything I could, but I still got it. (RN)
Q12 With all our risks it’s ridiculous because everyone has rules. It depends on who you work with. I know how to get masks, but others don’t. I just get what I need. (RN)
Q13 I care for my residents. They don’t have anyone. I had to hold the hands of 2 of my residents when they were dying. That was hard. (CNA)

Note. RN, registered nurse; CNA, certified nursing assistant; LPN, licensed practical nurse.

The acute shortage of personal protective equipment (PPE) has been disproportionately challenging for nursing homes compared to hospitals. The rise in hospitalized patients initially led to PPE guidelines being more “hospital-centric,”^3^ with nursing homes adapting those guidelines. For example, prior to the pandemic, most frontline nursing home HCWs did not routinely use respirators such as N95 masks. During the pandemic, nursing homes were unable to rapidly fit test their HCWs for these masks. As one certified nursing assistant (CNA) trainee said, “It [N-95 mask] was cutting into my skin and was the wrong size. But they said they only had 1 size and I had to wear it.” With limited PPE supplies, HCWs were given 1 surgical mask per week, which felt “like fighting a war with rocks while the other guys [coronavirus] have guns.” HCWs shared gowns, used raincoats as gowns, or wore ill-fitting gowns (Table 1, Q5). Additionally, the messaging around PPE use was chaotic, and protocols changed frequently (Table 1, Q6 and Q7). HCWs had limited access to PPE and needed to follow cumbersome rules for procurement, leading some HCWs to purchase masks from “beauty salons” and “grabbing 2 masks instead of 1 when no one is there.”

Physically stressed and emotionally vulnerable, our participants were scared to “have to go back there.” During the severe acute respiratory syndrome epidemic in 2002, HCWs experienced distress due to perceived stigma and fear of contagion.^4^ Lack of support and stigma contribute to distress, and those at higher risk for exposures experience greater post-traumatic stress disorder.^5^ Although the foremost fear is fear of developing COVID-19 and transmitting SARS-CoV-2,^6^ our participants were also afraid of not knowing how they got infected, of reinfection, and of transmitting the virus to their families (Table 1, Q8, Q9, and Q10). They felt their workplaces had failed them, and they were angered that, despite the risks, they were not protected (Table 1, Q11). They perceived disparities related to PPE when they saw nursing home administrators had N95 masks while “those in the front line [were] not protected” (Table 1, Q12).

Although their counterparts in hospitals were regaled as heroes, nursing home HCWs felt ignored, overwhelmed, stigmatized, and underprepared to cope physically and emotionally. An LPN said, “It messes with me mentally. I am just scared that I don’t know anything. I’ve been a nurse for 28 years and I *thought* I was so careful.” Some were burdened by the guilt of having unknowingly transmitted the infection to their residents. With restrictions on visitors and limited staff, several HCWs experienced acute grief watching the residents they had loved die (Table 1, Q13).

Despite physical exhaustion, anxiety, anger, guilt, and grief, these HCWs were the unsung heroes who continued to serve vulnerable older adults.^3^ It has been recommended that healthcare facilities prepare for pandemics by building individual and organizational resilience and by providing training and support to staff.^4^ Although COVID-19 is described as an “occupational disease” for which HCWs need social and psychological support,^7^ our participants felt unsupported and feared returning to work.

Nursing homes need more resources, and COVID-19 may not be the last challenge of this magnitude that we will face. Therefore, it is imperative that nursing home HCWs are supported through measures such as (1) education and training on pandemics and disaster preparedness, (2) easy access and clear communication on infection control and appropriate use of PPE, (3) systems to cope physically under such circumstances such as rest breaks, and (4) systems to cope emotionally such as training on caring for the dying, and grief counseling to cope with death. With the continuous rise in the number of vulnerable older adults needing long-term care and the decrease in staff willing and needed to care for them, these measures may no longer be optional.
